# Biomechanical properties of different anterior and posterior techniques for atlantoaxial fixation: a finite element analysis

**DOI:** 10.1186/s13018-023-03905-3

**Published:** 2023-06-26

**Authors:** Jie Li, Shuai Cao, Dong Guo, Teng Lu, Quanjin Zang

**Affiliations:** 1 Department of Orthopedics, The Second Affiliated Hospital of Xi’an Jiaotong University, 157Th West Fifth Road, Xi’an, 710004 Shaanxi Province China; 2 Department of Orthopedics, Civil Aviation General Hospital, No. 1, Gaojing Stress, Chaoyang District, Beijing, 100123 China

**Keywords:** Atlantoaxial fixation, Nonfixed segment degeneration, Segmental stability, Stress concentration, Finite element

## Abstract

**Background:**

Many techniques for atlantoaxial fixation have been developed. However, the biomechanical differences among various atlantoaxial fixation methods remain unclear. This study aimed to evaluate the biomechanical influence of anterior and posterior atlantoaxial fixation techniques on fixed and nonfixed segments.

**Methods:**

An occiput-C7 cervical finite element model was used to construct 6 surgical models including a Harms plate, a transoral atlantoaxial reduction plate (TARP), an anterior transarticular screw (ATS), a Magerl screw, a posterior screw-plate, and a screw-rod system. Range of motion (ROM), facet joint force (FJF), disc stress, screw stress, and bone-screw interface stress were calculated.

**Results:**

The C1/2 ROMs were relatively small in the ATS and Magerl screw models under all loading directions except for extension (0.1°–1.0°). The posterior screw-plate system and screw-rod system generated greater stresses on the screws (77.6–1018.1 MPa) and bone-screw interfaces (58.3–499.0 MPa). The Harms plate and TARP models had relatively small ROMs (3.2°–17.6°), disc stress (1.3–7.6 MPa), and FJF (3.3–106.8 N) at the nonfixed segments. Changes in disc stress and FJF of the cervical segments were not consistent with changes in ROM.

**Conclusions:**

ATS and Magerl screws may provide good atlantoaxial stability. The posterior screw-rod system and screw-plate system may have higher risks of screw loosening and breakage. The Harms plate and TARP model may more effectively relieve nonfixed segment degeneration than other techniques. The C0/1 or C2/3 segment may not be more susceptible to degeneration than other nonfixed segments after C1/2 fixation.

## Background

The atlantoaxial complex is the most mobile segment of the spine, and its range of motion (ROM) in rotation accounts for approximately 50% of the entire cervical spine rotation [1]. Atlantoaxial instability or dislocation caused by trauma, tumours, infection, and congenital malformation may lead to profound neurologic deficits and even death [2]. The goal of surgery should be to achieve reduction, decompression, fixation, and fusion of the atlantoaxial articular. The current approaches used to treat atlantoaxial instability are anterior and posterior atlantoaxial fixation techniques, such as transoral atlantoaxial plate fixation (Harms plate), transoral atlantoaxial reduction plate (TARP), anterior transarticular screw (ATS), posterior transarticular screw (Magerl screw), posterior screw-plate system, and posterior screw-rod system [3–5].

Currently, studies have demonstrated that posterior surgery represented by C1 lateral mass-C2 pedicle screw fixation achieves reliable stability and has high fusion rates and excellent clinical outcomes, so it has become the commonly used method for atlantoaxial fixation [6–8]. However, the posterior approach may present unsatisfactory decompression and reduction results in some cases in addition to the risk of vertebral artery injury and causing more extensive soft tissue damage than the anterior approach [9, 10]. In particular, the anterior approach is suitable for patients with a vertebral artery with an abnormal course and patients with no posterior bony structure [11]. To date, many in vitro and finite element studies have investigated the biomechanical differences in atlantoaxial ROM and implant stress between various posterior fixation techniques [12–14]. Conversely, anterior fixation techniques have been less studied to date, and concerns are often raised about the biomechanical behaviour of the atlantoaxial joint. In addition, these studies showed heterogeneity due to anatomical differences, operative procedures, and loading conditions. Thus, these results should be cautiously interpreted.

In addition, it is well known that nonfixed segment degeneration, especially adjacent segment degeneration, is one of the most common long-term complications of cervical arthrodesis, which can provoke typical neck pain [15, 16]. Numerous studies have demonstrated that segment degeneration is closely related to abnormal increases in ROM, disc stress, and facet joint force (FJF) at nonfixed segments [17–19]. However, the influence of different atlantoaxial fixation techniques on nonfixed segments remains unclear. Notably, previous finite element studies simulating atlantoaxial fixation only considered the reconstruction of the upper cervical spine rather than the whole cervical spine [20–22]. It is necessary to establish finite element models of the whole cervical spine to better understand how the ROM, disc stress, and FJF change with respect to different fixation techniques and loading conditions for cervical segments. This may be beneficial for understanding the occurrence of segment degeneration after atlantoaxial fixation.

Finite element analysis based on numerical models is widely used in biomechanical research since it allows us to easily investigate joint mobility and stress distribution. To evaluate the biomechanical influence of various atlantoaxial fixation techniques on fixed and nonfixed segments, an occiput (C0)–C7 cervical finite element model was constructed, and six surgical models were developed (the Harms plate, TARP, ATS, Magerl screw, screw-plate system, and screw-rod system). The ROM, FJF, and maximum stresses on the screws, bone-screw interfaces, and intervertebral discs were calculated and analysed.

## Methods

### Modelling of the intact cervical spine

In this study, a finite element model of the C0–C7 cervical spine was constructed. Informed consent for the use of individual data was obtained from the participant. First, the cervical computed tomography scans (1.0 mm thickness) of a healthy male (30 years of age; height, 176 cm; weight, 60 kg) without any cervical spine deformity or related diseases were imported into Mimics 21.0 software (Materialise, Leuven, Belgium). Then, the rough geometric model of the cervical vertebrae was generated by executing a series of software commands, such as threshold segmentation and regional growth. For further correction, surface tuning and optimization with 3-Matic 11.0 (Materialize, Leuven, Belgium) was used to build a geometric solid model of the bone, cartilage, and intervertebral discs. Next, HyperMesh 2019 (Altair Engineering, Inc., Troy, Michigan, USA) was used to mesh the model and construct the major ligaments. Finally, model assembly, material property definitions, and finite element analysis were performed using Abaqus 6.13 (Dassault System, Paris, France).

**Fig. 1:**
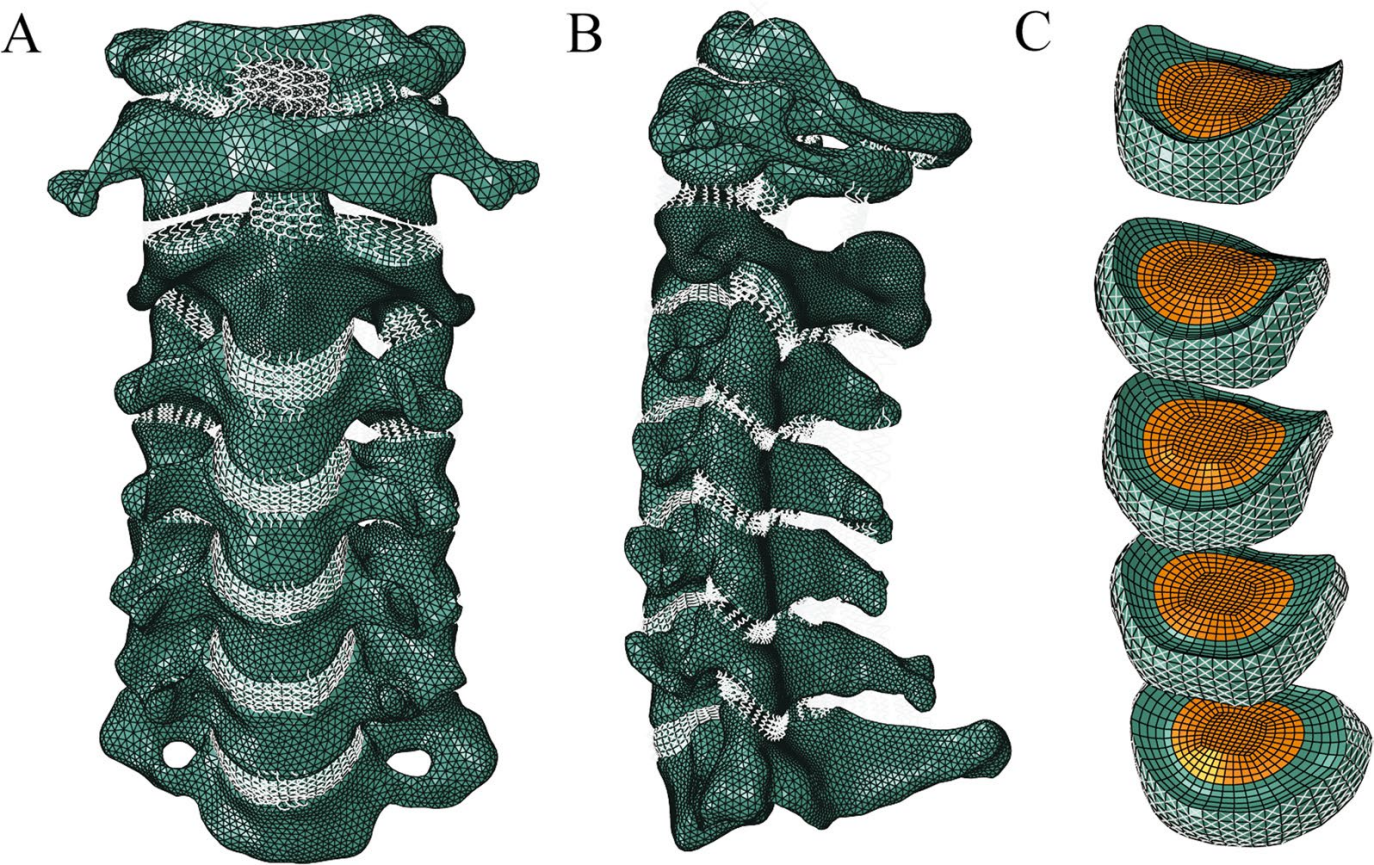
The intact finite element model of the cervical spine. **A** Front view. **B** Lateral view. **C** Intervertebral disc

The intact model consisted of the occiput, 7 cervical vertebrae, 5 intervertebral discs, and ligaments (Fig. 1). The thicknesses of the cortical bone and cartilage endplates were 1 mm and 0.5 mm, respectively. The intervertebral disc was composed of the nucleus pulposus, annulus fibres, and annulus ground substance. The fibres were embedded in the annulus ground substance with an inclination of ± 30°–45°.

**Fig. 2:**
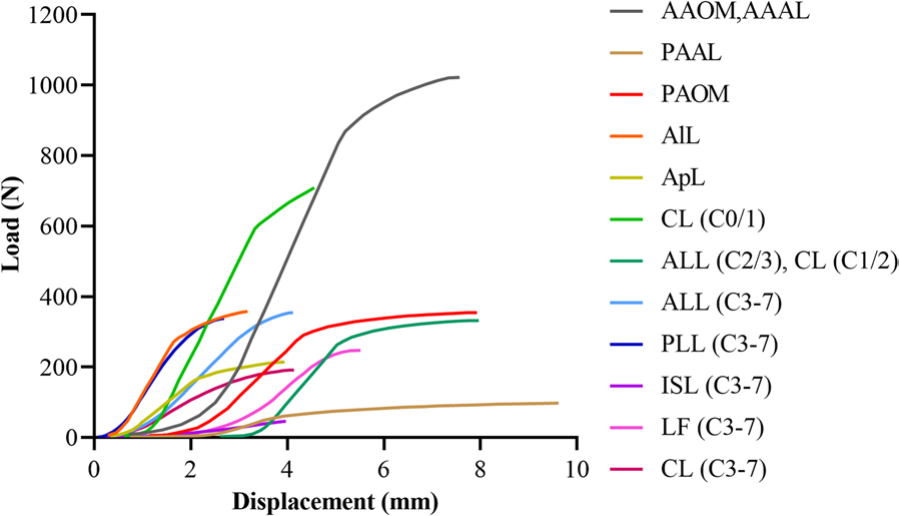
Ligament load‒displacement curve in the finite element model. (AAOM, anterior atlantooccipital membrane; AAAL, anterior atlantoaxial ligament; PAAL, posterior atlantoaxial ligament; PAOM, posterior atlantooccipital membrane; AlL, alar ligament; ApL, apical ligament; ALL, anterior longitudinal ligament; PLL, posterior longitudinal ligament; ISL, interspinous ligament; LF, ligamentum flavum; CL, capsular ligament)

The spinal ligaments included the tectorial membrane, transverse ligament, apical ligament, alar ligament, anterior atlantooccipital membrane, anterior atlantoaxial ligament, posterior atlantooccipital membrane, posterior atlantoaxial ligament, anterior longitudinal ligament, posterior longitudinal ligament, ligamentum flavum, interspinous ligament, and capsular ligament. The tectorial membrane and transverse ligament were modelled using 4-node membrane elements. All other ligaments were represented with nonlinear tension-only spring elements (Fig. 2).

Hyperelastic materials were applied to the cartilage, nucleus pulposus, and annulus ground substance. The annulus fibres were defined as hypoelastic material properties. Frictionless soft contact was applied to simulate all the contact interactions, including the facet joints, atlantooccipital joint, atlantoaxial joint, and odontoid joint. The cortical bone and cancellous bone were simulated as isotropic elastic materials. The occiput was simulated as a rigid body. A convergence analysis was performed to ensure that the maximum changes in the strain energy were < 5%. The element types and material properties referred to previous studies, as shown in Table 1 [23].

**Table 1:** Material properties and element types in the finite element models

Materials	Element type	Young’s modulus (MPa)	Poisson’s ratio (μ)
Cortical bone	C3D4	12,000	0.3
Cancellous bone	C3D4	450	0.2
Joint cartilage	C3D8I	10	0.3
Cartilage endplate	C3D8I	24	0.4
Nucleus pulposus	C3D8H	1	0.49
Annulus ground	C3D8H	4.2	0.45
Annulus fibre	T3D2	450	0.3
Tectorial membrane	4-node membrane elements	20	0.3
Transverse ligament		20	0.3
Implants	C3D4	110,000	0.3

### Modelling of the surgical procedures

**Fig. 3:**
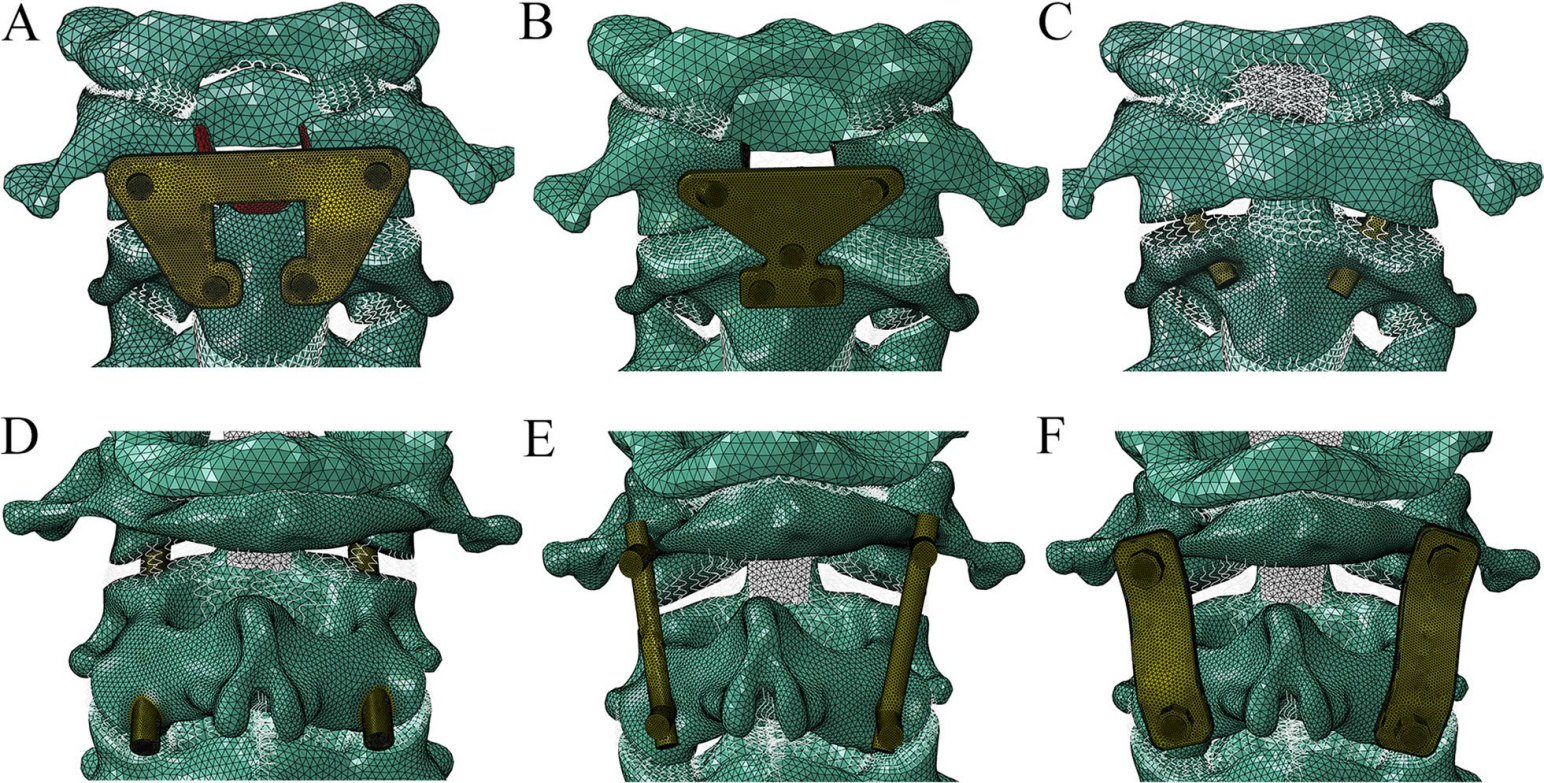
The surgical models for atlantoaxial fixation. **A** Transoral atlantoaxial reduction plate. **B** Harms plate. **C** Anterior transarticular screw. **D** Magerl screw. **E** Screw-rod system. **F** Screw-plate system

Six surgical models were developed based on the intact model to simulate anterior and posterior atlantoaxial fixation. Anterior fixation techniques were performed using the Harms plate, TARP, and ATS. Specifically, the anterior arch of C1, the odontoid process, and the ligaments associated with them were removed to allow placement of the TARP and Harms plate. A butterfly shaped TARP with a thickness of 2 mm was fixed with bilateral anterior C1 lateral mass screws and C2 vertebral body screws (Fig. 3A). A Harms plate was fixed with two atlantal screws and three axial screws (Fig. 3B). For ATS fixation, the anterior transarticular screws were advanced into the C2 body from the undersurface of the overhanging lip of the lateral mass of C2 and 5 mm lateral to the base of the odontoid process, passed through the midpoint of the atlantoaxial joint and entered the C1 lateral mass (Fig. 3C). Posterior fixation techniques included the Magerl screw, screw-plate system, and screw-rod system. In the Magerl technique, two transarticular screws were inserted from the C2 inferior articular process, passed across the atlantoaxial lateral joints and directed towards the C1 anterior arch (Fig. 3D). For the posterior screw-rod system, four independent screws were inserted into the bilateral C1 lateral mass and the C2 pedicle, and the ipsilateral C1 and C2 screws were connected by a rod (Fig. 3E). The screw position of the posterior screw-plate system was the same as that of the screw-rod system, and the ipsilateral C1 and C2 screws were connected by a plate (Fig. 3F).

The surgical models were performed as reported previously in the literature [24–29]. The diameter of the screws and rods was 3.5 mm. The lengths of the C1 lateral mass screws, C2 pedicle screws, C2 vertebral body screws, anterior transarticula screws, and posterior transarticula screws were 30 mm, 30 mm, 10 mm, 20 mm, and 40 mm, respectively. All implant components were designed as titanium alloys. The bone–screw, screw-plate, and screw–rod interfaces were defined as a tied contact condition.

### Loading and boundary conditions

The lower surface of the C7 vertebra was constrained in all directions. To validate the intact model, a pure moment of 1.5 Nm was applied on the superior surface of the C0 to produce flexion, extension, lateral bending, and axial rotation. The C0–C7 ROMs and load‒deflection curves were compared with previously reported data. Then, a 1.5 Nm moment and 73.6 N follower load were applied to the intact model to determine the C0–C7 ROMs. The 73.6 N follower load was used along the physiological curvature of the cervical spine to simulate the head weight and muscle force [18]. Displacement control was performed for the surgical models to ensure that their C0–C7 ROMs were the same as those of the intact model. The ROM, FJF, screw stress, bone-screw interface stress, and disc stress of different models were analysed. The FJF was recorded from both the left and right facets and averaged for each level during extension. For axial rotation and lateral bending, only the forces from the loaded facets were used.

## Results

### Model validation

**Fig. 4:**
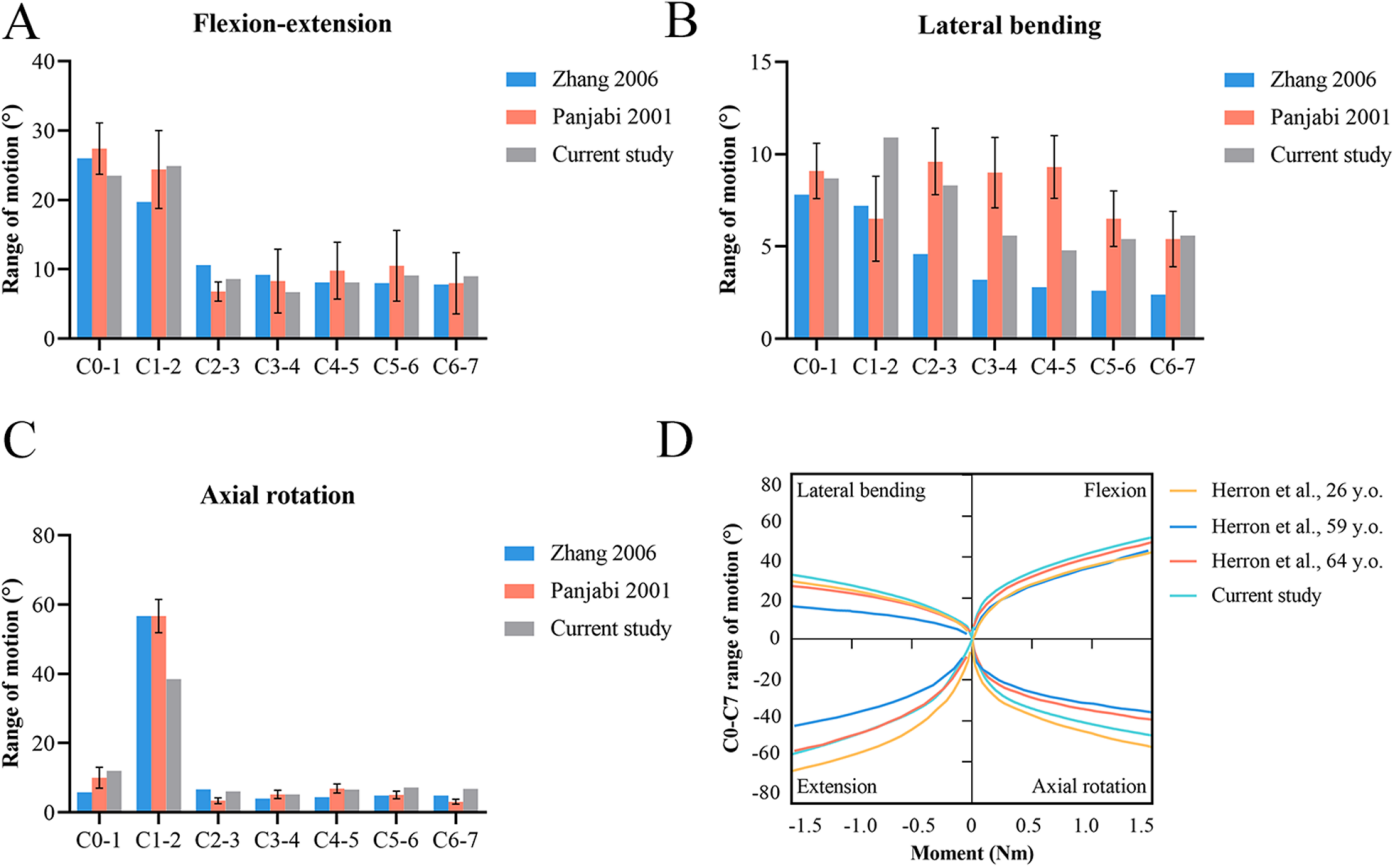
Validation of the intact model. Intersegmental range of motion under a pure moment of 1.5 Nm: **A** flexion–extension, **B** lateral bending, and **C** axial rotation. **D** Load‒deflection curves

Under flexion–extension, lateral bending, and axial rotation, the ROM of each segment was compared with the previous finite element study by Zhang et al. in 2006 and in vitro experimental study by Panjabi et al. in 2001 [30, 31]. The ROMs of the current and previous studies were consistent (Fig. 4). The load‒deflection curves showed that the ROM increased nonlinearly with increasing moment, which was also in accordance with the existing results of studies by Herron et al. [32]. Therefore, the current model was considered reliable and could be used for further studies.

### The fixed segment

**Fig. 5:**
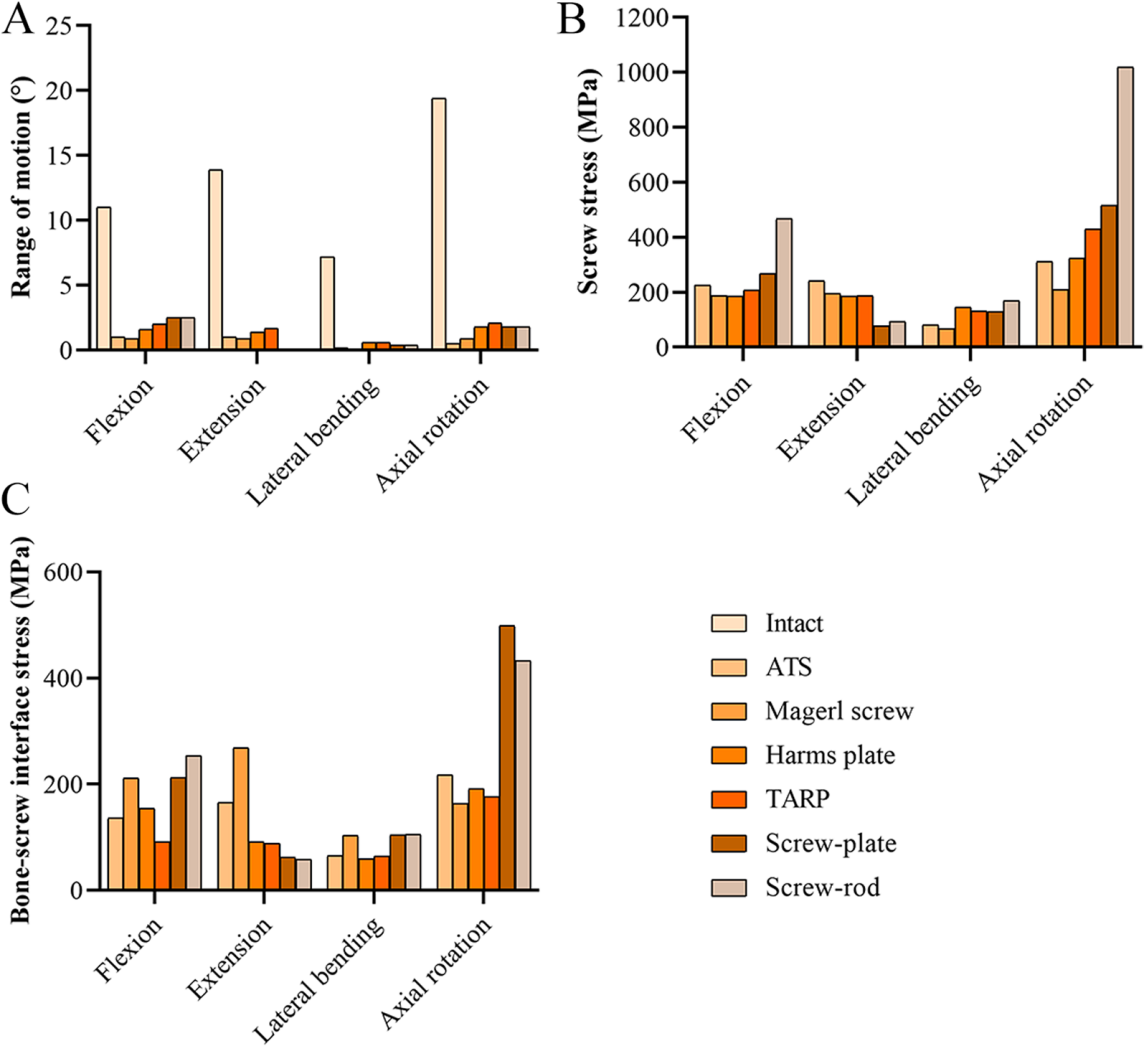
The range of motion and maximum stress at the fixed segment. **A** Range of motion. **B** Screw stress. **C** Bone-screw interface stress

In general, the C1/2 ROMs of all the surgical models (0.1°–2.5°) were significantly lower than that of the intact model (7.2°–19.4°) under all loading conditions (Fig. 5A). In the ATS, Magerl screw, Harms plate, TARP, screw-plate, and screw-rod models, the C1/2 ROM was 1.0°, 0.9°, 1.6°, 2.0°, 2.5°, and 2.5° under flexion, respectively; 1.0°, 0.9°, 1.4°, 1.7°, 0.1°, and 0.1° under extension, respectively; 0.2°, 0.1°, 0.6°, 0.6°, 0.4°, and 0.4° under lateral bending, respectively; and 0.5°, 0.9°, 1.8°, 2.1°, 1.8°, and 1.8° under axial rotation, respectively. For each surgical model, the maximum stress of the screws was induced during axial rotation. In the ATS, Magerl screw, Harms plate, TARP, screw-plate, and screw-rod models, the maximum stresses of the screws were 81.7–311.1 MPa, 68.0–210.1 MPa, 145.9–324.5 MPa, 133.0–429.2 MPa, 77.6–515.5 MPa, and 94.1–1018.1 MPa, respectively (Fig. 5B); the maximum stresses of the bone-screw interfaces were 65.3–217.7 MPa, 103.3–269.1 MPa, 59.8–191.4 MPa, 64.5–176.7 MPa, 62.0–499.0 MPa, and 58.3–433.0 MPa, respectively (Fig. 5C).

### The nonfixed segments

**Fig. 6:**
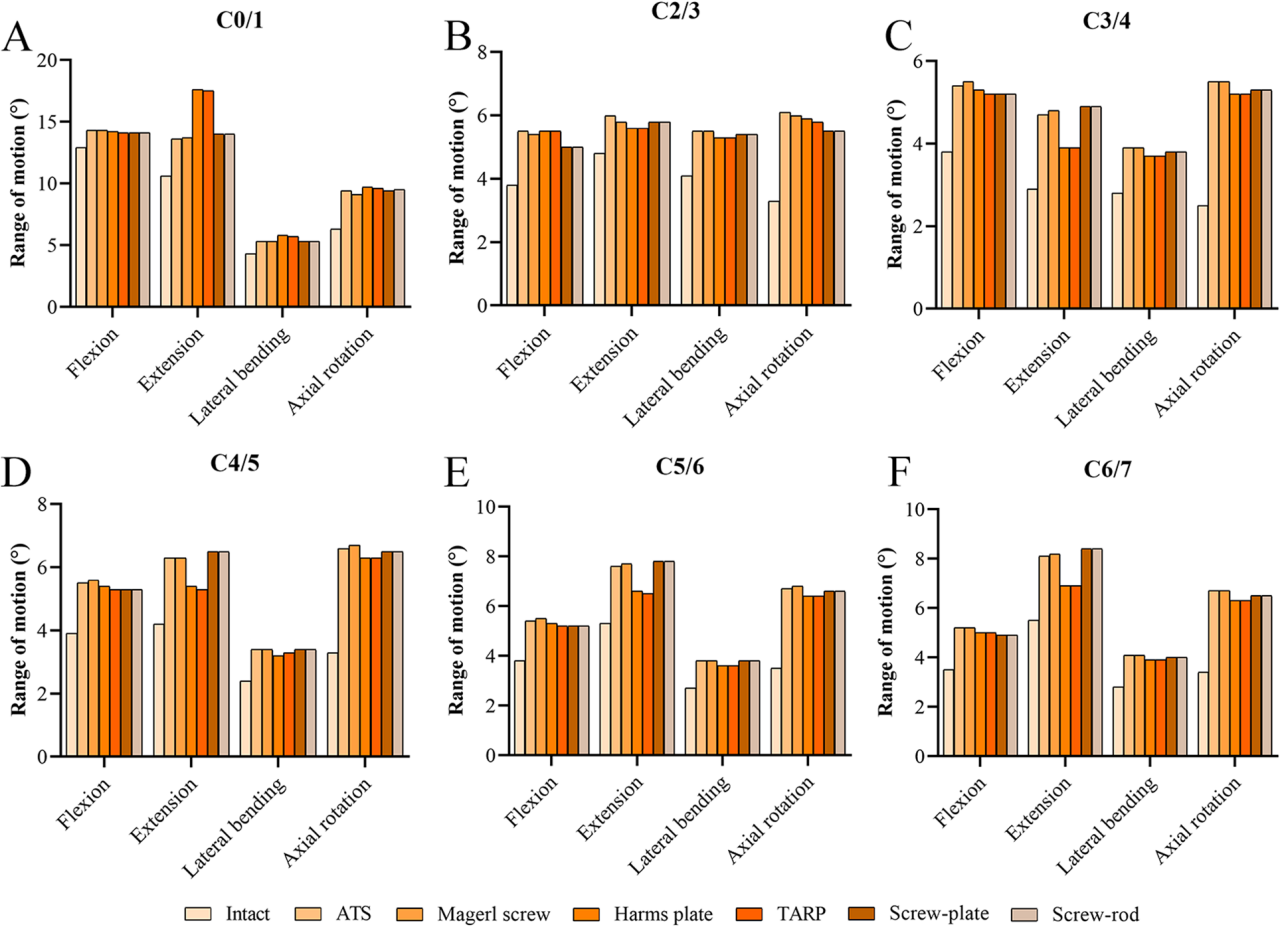
The range of motion at the nonfixed segments. **A** C0/1 segment. **B** C2/3 segment. **C** C3/4 segment. **D** C4/5 segment. **E** C5/6 segment. **F** C6/7 segment

For all the surgical models, the ROMs of the nonfixed segments (3.2°–17.6°) were higher than those obtained for the intact model (2.4°–12.9°), especially during axial rotation (Fig. 6). In the ATS, Magerl screw, Harms plate, TARP, screw-plate, and screw-rod models, the ROMs at the nonfixed segments were 3.4°–14.3°, 3.4°–14.3°, 3.2°–17.6°, 3.3°–17.5°, 3.4°–14.1°, and 3.4°–14.1°, respectively. The differences in the ROMs of each segment were very small among the six surgical models during flexion, lateral bending, and axial rotation. Under extension, the Harms plate and TARP models had relatively larger ROMs at the C0/1 segment (17.5°–17.6°) and lower ROMs at the C3–C7 segments (3.9°–6.9°).

**Fig. 7:**
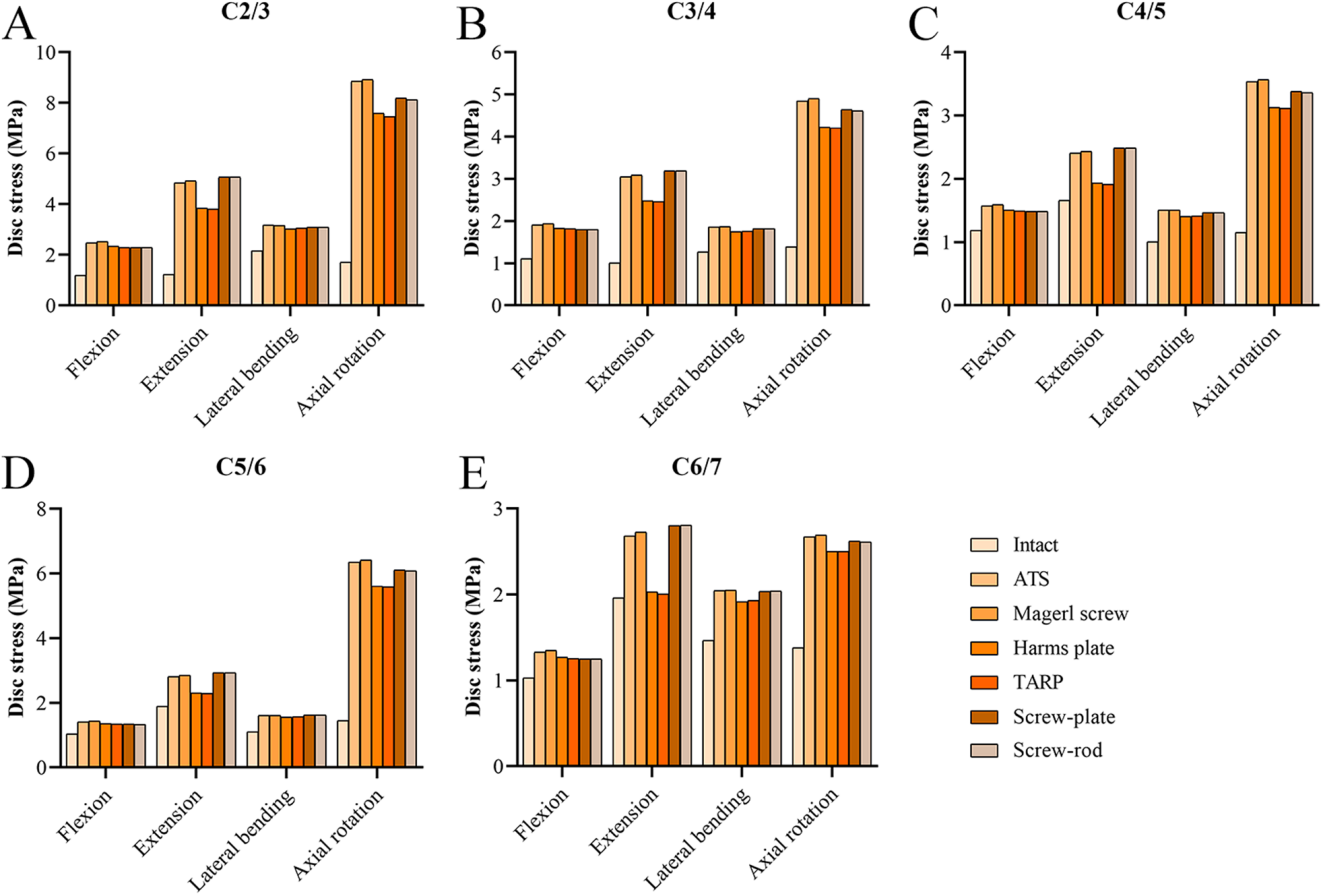
The disc stress at the nonfixed segments. **A** C2/3 segment. **B** C3/4 segment. **C** C4/5 segment. **D** C5/6 segment. **E** C6/7 segment

Compared with the intact model (1.0–2.2 MPa), all the implants increased the maximum disc stresses at nonfixed levels during all motions (1.2–8.9 MPa), especially during axial rotation (Fig. 7). In the ATS, Magerl screw, Harms plate, TARP, screw-plate, and screw-rod models, the maximum disc stresses were 1.3–8.9 MPa, 1.3–8.9 MPa, 1.3–7.6 MPa, 1.3–7.5 MPa, 1.3–8.2 MPa, and 1.2–8.1 MPa, respectively. The disc stresses in flexion and lateral bending were similar across all surgical models, whereas relatively smaller disc stresses in the Harms plate and TARP models were noted in extension and axial rotation.

**Fig. 8:**
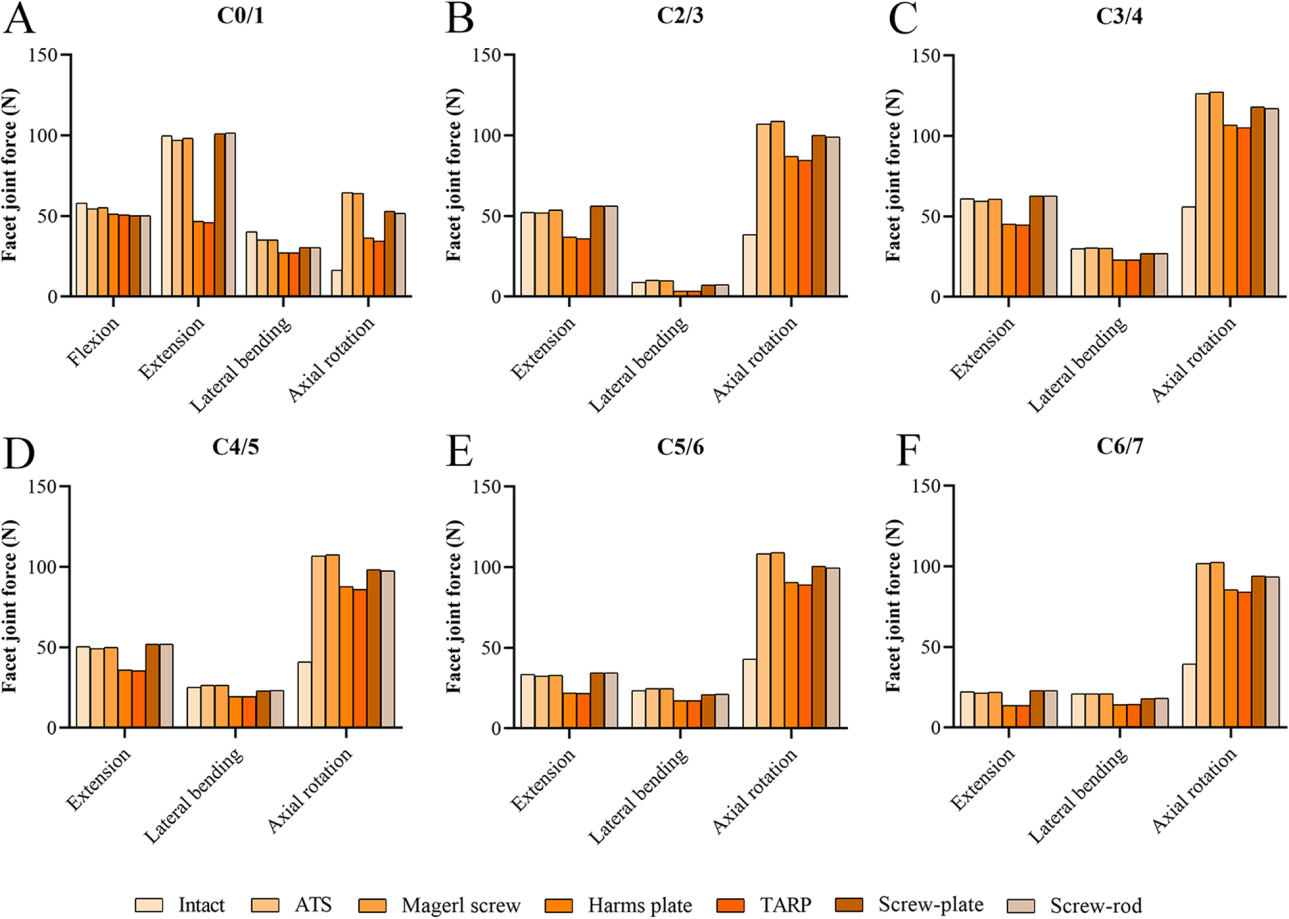
The facet joint force at the nonfixed segments. **A** C0/1 segment. **B** C2/3 segment. **C** C3/4 segment. **D** C4/5 segment. **E** C5/6 segment. **F** C6/7 segment

FJF was not detected in each segment of the intact or surgical models during flexion except for the C0/1 segment. Compared with the intact model (16.4–55.9 N), the FJF at each level in all surgical models (34.6–127.4 N) increased substantially during axial rotation (Fig. 8). In the ATS, Magerl screw, Harms plate, TARP, screw-plate, and screw-rod models, the FJF was 10.1–126.4 N, 9.9–127.4, 3.4–106.8 N, 3.3–105.2 N, 7.2–118.0 N, and 7.3–117.1 N, respectively. The FJF for the Harms plate and TARP was the smallest among the six surgical models during all motions and was smaller than that for the intact model during extension and lateral bending.

### Changes in ROM, disc stress, and FJF with intervertebral levels

**Fig. 9:**
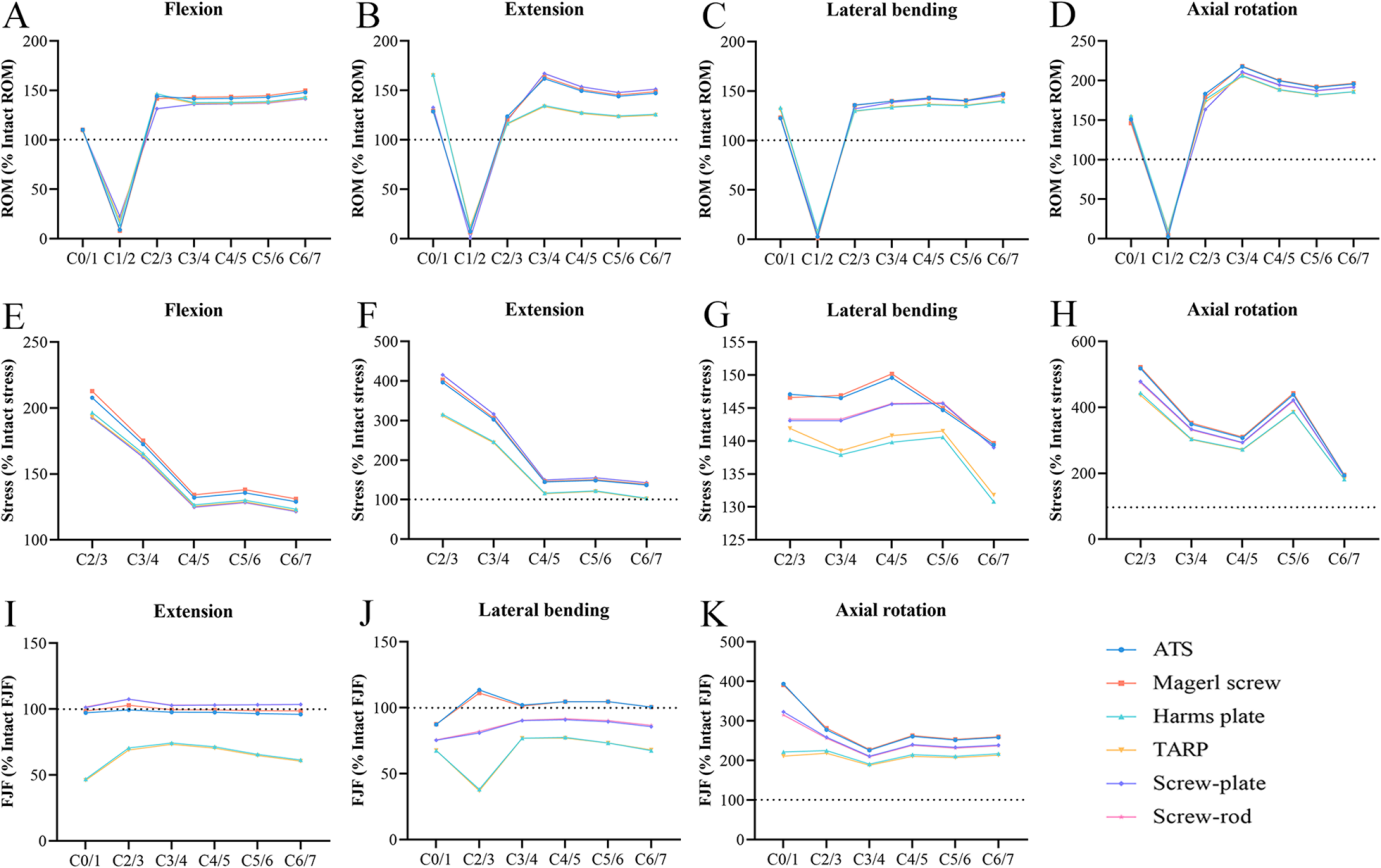
Changes in ROM, disc stress, and FJF with intervertebral levels. ROM: **A** Flexion. **B** Extension. **C** Lateral bending. **D** Axial rotation. Disc stress: **E** Flexion. **F** Extension. **G** Lateral bending. **H** Axial rotation. FJF: **I** Extension. **J** Lateral bending. **K** Axial rotation. (ROM, range of motion; FJF, facet joint force)

The results of the ROM, disc stress, and FJF were normalized with respect to the intact model (Fig. 9). At the C0/1, C1/2, C2/3, C3/4, C4/5, C5/6, and C6/7 levels, the ROMs were 109.1–166.1%, 0.7–22.7%, 116.0–183.3%, 133.5–218.2%, 126.7–200.4%, 123.4–192.1%, and 125.0–196.5%, respectively. At the C2/3, C3/4, C4/5, C5/6, and C6/7 levels, the disc stresses were 140.2–522.0%, 137.9–352.6%, 115.3–310.0%, 121.1–442.9%, and 102.3–195.0%, respectively. At the C0/1, C2/3, C3/4, C4/5, C5/6, and C6/7 levels, the FJF was 46.0–393.9%, 37.1–281.9%, 73.3–227.9%, 70.4–263.1%, 64.8–253.5%, and 60.5–260.2%, respectively.

## Discussion

Due to the deep anatomical position and frequent variation of the vertebral artery, atlantoaxial fixation is challenging, risky and was once avoided in spinal surgery. Since sublaminar wires were first proposed for atlantoaxial fixation by Gallie et al. in 1939, researchers have continuously improved posterior fixation techniques by using Magerl transarticular screws, C1 lateral mass screws, C2 pedicle screws, and C2 translaminar screws to meet the needs of individual patients. In addition, anterior atlantoaxial fixation with the Harms plate, ATS, and TARP that was developed in the 1980s has improved surgical strategies. Despite this, when planning atlantoaxial fixation, particularly anterior fixation, decisions must be made carefully to avoid problems with internal fixation stability, including screw breakage or loosening, and nonfixed segment degeneration. To this end, six typical anterior and posterior surgical models were constructed in this study to comprehensively evaluate the biomechanical differences between different models.

One of the primary objectives of surgery is to achieve rigid fixation of the atlantoaxial articulation, and ROM is the most important index for evaluation of segmental stability. In this study, all surgical models significantly limited the ROMs of the C1/2 segment in all directions when compared with the intact model. Interestingly, there are only slight differences among surgical models. In particular, there was almost no difference between the ATS and Magerl screws, between the Harms plate and TARP, and between the posterior screw-rod system and screw-plate system. This may be because their design concepts based on biomechanical mechanisms were similar. Moreover, studies have shown that the C1/2 segment is responsible for 63%-73% of the rotational movement of the cervical spine, while only 12% contributes to flexion–extension, so limiting atlantoaxial rotation is essential [33]. Here, it was found that the ROM of the C1/2 segment was relatively small in the ATS and Magerl screw models during all motions except extension. One reason may be due to the position of the transarticular screw near the centre of rotation of the C1/2 segment. From this point of view, the transarticular screw that is used in anterior or posterior approaches showed superior performance, which was also consistent with results from Erbulut et al. [12]. Previous cadaveric studies found that Magerl screws provided excellent stability for lateral bending and axial rotation and that wiring achieved a three-point fixation, thereby achieving the highest stability [34]. A recent study by Thomas et al. indicated that Magerl screw fixation without a supplementary posterior construct would provide sufficient stability and achieve satisfactory clinical outcomes in patients with atlantoaxial instability caused by rheumatoid arthritis [35]. However, a finite element analysis conducted by Chun et al. showed that the Magerl screw was less effective for stabilization than the C1 lateral mass-C2 pedicle screw [6]. The heterogeneity in modelling methods and loading conditions could explain the difference in the study results. Although the fusion rate of Magerl screws is 100%, thereby indicating good stability, approximately 22% of patients are unsuitable for Magerl screw placement due to some anatomical factors [36]. Given the relatively small risks of vertebral artery and spinal cord injuries during screw placement, ATS may be a viable option when Magerl screws are not feasible.

When evaluating structural stability, not only the ROM of the segment but also the stress concentration effect of the implants should be considered because of extensive cyclic loading imposed on the highly mobile upper cervical region. Under sustained mechanical loading, instrumentation failure due to screw loosening or breakage is likely to occur, which seriously affects the long-term stability of surgical segments. Our study investigated the maximum von Mises stresses of the screws and bone-screw interfaces in each surgical model. The findings revealed that the posterior screw-rod system and the screw-plate system generated greater stresses on screws and bone-screw interfaces during all movements except for extension, suggesting that they may have higher risks of screw loosening and breakage. We speculated that this may be attributed to the load distribution of the cervical spine, with 36% in the anterior column and 64% in the posterior columns [37]. Kim et al. reported that the rate of screw fracture was 7.4% (2/27) for screw-rod constructs and 7.1% (1/14) for C1-2 transarticular screws [38]. For TARP fixation, no screw breakage has been reported thus far, and only a few cases of screw loosening have been reported in the literature. Nevertheless, the reliability of these clinical studies was affected by sample size, follow-up time, age, bone mineral density, screw position, and other factors. Thus, additional research is required in the future. It is worth noting that Harms plates are more likely to incur screw loosening in clinical practice due to the absence of a screw-locking mechanism [26]. However, the contact modes of all screw-plate and bone-screw interfaces were simplified in our study; therefore, it is not surprising that the maximum stresses of screws and bone-screw interfaces in the Harms plate model were similar to those in the TARP model.

Until now, the origin of nonfixed segment degeneration has remained controversial. However, overwhelming evidence suggests that it is associated with abnormal alterations in mechanical loading and a compensatory increase in ROM. Consistently, it was noticed that the ROMs and disc stresses of nonfixed segments increased in surgical models under all loading directions compared to the intact model. After atlantoaxial fixation, the FJF of each nonfixed segment mainly increased during rotation. Among the six surgical models, a relatively small ROM, disc stress, and FJF were observed in the models with Harms plate and TARP, indicating that they may delay the progression of degeneration. Harms plates are seldom used in clinical applications because they are prone to screw loosening and other adverse events. Yin et al. developed the TARP system in 2002, which allowed operators to perform decompression, reduction, internal fixation, and fusion through the single transoral approach in a one-stage operation without an additional posterior procedure [39]. To date, this technology has been improved to the fourth generation, and clinical studies have shown a favourable outcome for patients who were treated with TARP. Finally, we sought to understand how the ROM, disc stress, and FJF changed with cervical levels. Interestingly, we found that the changing trends of disc stress and FJF were not consistent with those of ROM. Moreover, the ROM compensation did not demonstrate a smooth decreasing trend with the increase in the distance from the surgical segment. Thus, the C0/1 or C2/3 segment may not be more susceptible to degeneration than other nonfixed segments after C1/2 fixation. This is possibly related to the anatomical characteristics of intervertebral discs and facet joints of the cervical spine. In short, the effects of different atlantoaxial fixation techniques on nonfixed segment degeneration need to be further confirmed.

This study has some limitations. First, the screws, ligaments, and loading conditions were simplified, and the impacts from surrounding muscles were ignored in the model. Thus, a gap may exist between the finite element model and a real spine system, and the results need to be further evaluated and verified by more high-quality studies in the future. Second, six classic techniques of atlantoaxial fixation were simulated in this study, and more investigations are required to explore the other techniques. Finally, finite element models were constructed based on the CT data of a healthy male subject. The effect of anatomic structure, osteoporosis, and other factors on biomechanical properties was overlooked.

## Conclusion

In this study, the ATS and Magerl screws may provide good atlantoaxial stability under all loading directions except for extension. Compared to the other fixation techniques, the posterior screw-rod system and screw-plate system generated greater stresses on screws and bone-screw interfaces, suggesting that they may have higher risks of screw loosening and breakage. The Harms plate and TARP resulted in a relatively small ROM, disc stress, and FJF at the nonfixed segments, which indicated that they may delay the progression of segment degeneration. The C0/1 or C2/3 segment may not be more susceptible to degeneration than other nonfixed segments after C1/2 fixation.

## Data Availability

The datasets used during the current study are available from the corresponding author on reasonable request.

## Abbreviations

ROM: Range of motion
FJF: Facet joint force
TARP: Transoral atlantoaxial reduction plate
ATS: Anterior transarticular screw
